# Lockdown: A chance to strengthen the relationship or to widen the gap for tunisian couples?

**DOI:** 10.1192/j.eurpsy.2021.1738

**Published:** 2021-08-13

**Authors:** L.S. Meddouri, E. Cherif, A. Hajri, A. Maamri, H. Zalila

**Affiliations:** Psychiatry, Outpatient Service, Razi Hospital, Manouba, Tunisia

## Abstract

**Introduction:**

General lockdown due to the outbreak of Corona virus is a major change in people’s lives. Some mental health professionals consider it as a traumatic event with potentially serious psychiatric repercussions, especially on married couples.

**Objectives:**

Determine the consequences of the lockdown on married couples.

**Methods:**

An online survey conducted on social media during Mai 2020.

**Results:**

A total of 223 married persons filled our survey with 86,1% females. Marriage was traditional in 17%, through mutual friends and acquaintances in 0,4% and after a love story in 84,3%. Families were not consenting to the marriage in 5,4% and 28,7% of participants did not consider that they knew their partner enough prior to marriage. The mean duration of marriage was 2 years and 10 months. Having children was reported in 77,1%. Before the general lockdown, 1,79% described their relationship as “bad” compared to 5,82% during; sharing house chores raised from 56,44% to 68,44%. And taking care of children by both parents during the lockdown raised also from 55% to 67,22%. Frequency of sexual intercourse was the same in 57,4% and lower in 23,8%. The lockdown was an opportunity to discover new things in their partner for 28,4% and 19,7% did not consider the lockdown as a chance to spend more time with their partner.

**Conclusions:**

The lockdown gave us a chance to study the impact of the absence of social life on married couples.

**Disclosure:**

No significant relationships.

